# Ontogenetic feeding shifts in two thresher shark species in the Galapagos Marine Reserve

**DOI:** 10.7717/peerj.18681

**Published:** 2024-12-16

**Authors:** Camila Arnés-Urgellés, Felipe Galván-Magaña, Fernando R. Elorriaga-Verplancken, Antonio Delgado-Huertas, Diego Páez-Rosas

**Affiliations:** 1 Centro Interdisciplinario de Ciencias Marinas, Instituto Politécnico Nacional, La Paz, Baja California Sur, Mexico; 2 Galapagos Science Center, Universidad San Francisco de Quito, Isla San Cristóbal, Islas Galápagos, Ecuador; 3 Instituto Andaluz de Ciencias de la Tierra (CSIC-UGR), Universidad de Granada, Granada, Granada, Spain; 4 Fundación Conservando Galápagos, Galapagos Conservancy, Isla Santa Cruz, Islas Galápagos, Ecuador; 5 Oficina Técnica San Cristóbal, Direccion Parque Nacional Galápagos, Isla San Cristóbal, Islas Galápagos, Ecuador

**Keywords:** Feeding strategies, Isotopic niche, Ontogenetic changes, Tropical Eastern Pacific, Thresher sharks

## Abstract

**Background:**

The morphology and hunting behavior of thresher sharks make them easily distinguishable. These species are distributed across the Tropical Pacific Ocean feeding on squid and small fish. However, ontogenetic changes in their feeding strategies and habitat use are still unknown in this region.

**Methods:**

We examined the δ^13^C and δ^15^N signatures in vertebral collagen from populations of *Alopias pelagicus* and *Alopias superciliosus* inhabiting the Galapagos Marine Reserve, focusing on three maturity stages: neonate, juvenile and adult. The vertebrae samples were taken from the seizure of illegal fishing activities carried out by a foreign fleet within the Galapagos archipelago. A total of thirty-three vertebrae from *A. pelagicus* and twenty-one from *A. superciliosus* were analyzed.

**Results:**

Both species displayed significant differences in their δ^15^N values (*p* < 0.001), but not in δ^13^C (*p* = 0.230), suggesting a similar habitat use, but different prey consumption. Throughout their ontogeny, *A. pelagicus* displayed isotopic differences (*p* < 0.001), where neonates showed lower δ^13^C values and higher δ^15^N values compared to juveniles, probably because they still reflect the isotopic signatures of their mothers even after the first year of life. This study highlights trophic differences between both species, accompanied by an ontogenetic variation in *A. pelagicus*, aspects that allow us to understand the role of these species within the dynamics of the Eastern Tropical Pacific ecosystem.

## Introduction

Habitat utilization and movement of marine vertebrates are mainly driven by ontogenetic shifts in their life-history priorities, such as survival, growth, and reproduction (Heupel, Carlson & Simpfendorfer, 2007; Carlisle et al., 2015). This premise, coupled with intraspecific and interspecific interactions, influences marine trophic dynamics, ecosystem structure, and overall biodiversity (Werner & Gilliam, 1984; Baum & Worm, 2009). Thus, ontogenetic shifts have been studied in elasmobranchs because of their predatorial roles in most marine ecosystems (MacNeil, Skomal & Fisk, 2005; Estupiñán-Montaño et al., 2019). In recent years, these studies have emphasized the necessity of implementing science-based conservation management strategies (Shiffman et al., 2021; Cerutti-Pereyra et al., 2022), as large pelagic fish populations have increasingly decreased, along with the negative impacts of this phenomenon across numerous trophic levels (Baum & Myers, 2004; Ferretti et al., 2010; Bird et al., 2018).

Thresher sharks (*Alopias* spp) inhabit tropical and subtropical waters worldwide (Compagno, 1984). These species have an elongated dorsal lobe on their caudal fin, nearly as long as their body (Compagno, 1984), which is an essential part of their hunting behavior (Sepulveda et al., 2005; Oliver et al., 2013). The overexploitation of thresher sharks has threatened their survival (Worm et al., 2024), consequently, these species have been listed under Appendix II of the Convention on International Trade in Endangered Species of Wild Fauna and Flora (CITES). The International Union for Conservation of Nature (IUCN) red list assessment also classifies thresher shark populations as overexploited, leading to a decline in the global population (Rigby et al., 2019; Worm et al., 2024). Currently, in the Ecuadorian Pacific, thresher sharks represent more than 70% of the total shark catch (Raharjo, Hartati & Redjeki, 2024), even reaching catches exceeding 150,000 individuals over the last years (Briones-Mendoza, Mejía & Carrasco-Puig, 2022).

Despite this overexploitation, there is scarce knowledge about the life history of thresher sharks in the Eastern Tropical Pacific. A low number of studies have examined their feeding behavior in Ecuadorian waters (Polo-Silva et al., 2007; Polo-Silva, Rendón & Galván-Magaña, 2009; Polo-Silva et al., 2013; Páez-Rosas et al., 2018; Calle-Morán & Galván-Magaña, 2020). However, most of the research has focused on pelagic threshers (*Alopias pelagicus*), due to their higher landing quota, which facilitates for the collection of samples from fishing ports (Martínez-Ortíz et al., 2015; Briones-Mendoza, Mejía & Carrasco-Puig, 2022). Therefore, most of these studies have been conducted in mainland Ecuador, with the exception of Páez-Rosas et al. (2018), who based their research on pelagic threshers caught illegally in the Galapagos Marine Reserve (GMR). The GMR has some of the largest global shark aggregations (Salinas-de-León et al., 2016; Acuña-Marrero et al., 2018), and maintains relatively intact food webs that support the presence of these species (Páez-Rosas et al., 2024). Consequently, this region becomes an exceptional environment for shark research.

Stable isotope analysis (SIA) permits the quantification of changes in trophic interactions during different periods of development based on the isotopic turnover rate of the tissue (Bearhop et al., 2004; Hussey et al., 2012). Tissues with relatively low isotopic turnover rates (*e.g*., vertebral collagen) allow the inference of ontogenetic changes in foraging patterns in elasmobranchs (Estrada et al., 2006; Carlisle et al., 2015); since collagen is a metabolically inert tissue and is not resorbed after deposition (Campana, Natanson & Myklevoll, 2002). Therefore, successive layers of different density (*i.e*., annual groups of growth layers) reflect the conditions under which they were secreted (Campana, Natanson & Myklevoll, 2002), allowing to obtain a summary of feeding history and migratory behavior of these predators (Carlisle et al., 2015; Shen et al., 2022). This technique is based on the fact that isotopic signatures in consumer tissues are related to diet and habitat use (Michener & Schell, 1994; Bearhop et al., 2004). The δ^13^C values were assessed based on the principle that primary productivity, dissolved CO_2_ concentration, algal diversity, and other physicochemical processes create a pronounced coastal-oceanic gradient, resulting in a decrease in δ^13^C in offshore habitats (Michener & Schell, 1994; France, 1995; Newsome et al., 2007). And the principle that the δ^15^N of tissues increase as one ascends the trophic level owing to enrichment in the consumer’s δ^15^N relative to its prey (Adams & Sterner, 2000; Post, 2002).

Although ontogenetic studies of sharks in the GMR have been conducted (Estupiñán-Montaño et al., 2019; Salinas-de-León et al., 2019; Páez-Rosas et al., 2021; Cerutti-Pereyra et al., 2022), there is still a lack of understanding about the trophic ecology of sharks in this region. Therefore, this study aims to provide isotopic information differentiated by species, sex and maturity stages that allows understanding the feeding patterns of two thresher shark species (*A. pelagicus* and *A. superciliosus*) that inhabit the GMR. Furthermore, our work contributes to existing knowledge regarding the ecology of sharks in the Tropical Eastern Pacific and providing unprecedented insights into the trophic ecology of thresher sharks in the GMR.

## Materials and Methods

### Study area

**Figure 1:**
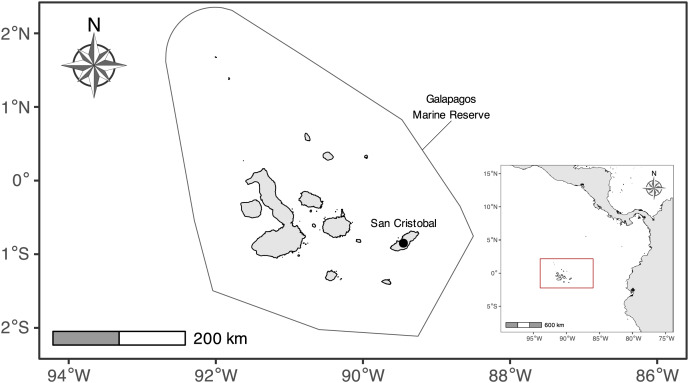
Map of the Galapagos Islands with the boundaries of the Galapagos Marine Reserve. The mark signals the location where the illegal fishing fleet was taken for inspection at San Cristobal Island.

The Galapagos Islands are in the Eastern Tropical Pacific Ocean, ~1,000 km from mainland Ecuador. This island complex is home to the GMR, which is limited by a strip of 40 nautical miles, measured from a “baseline” that surrounds the archipelago and its internal waters, generating a protected surface of ~138,000 km^2^ (Heylings, Bensted-Smith & Altamirano, 2002) (Fig. 1). Its remote location renders it susceptible to the impact of several oceanic currents, which, together with other environmental factors, contribute to their high endemism levels (Edgar et al., 2008; Salinas-de-León et al., 2020). The GMR is influenced by four primary oceanic currents from different directions (*i.e*., Perú, Panamá, Equatorial, and Sub-equatorial or Cromwell currents), which are responsible for important upwellings (Palacios et al., 2006; Schaeffer et al., 2008; Forryan et al., 2021), and the existence of a strong seasonality (Sweet et al., 2007).

### Sample collection

This research was undertaken under permits: PC-86-19 and was carried out following the protocols of ethics and animal handling approved by the Galapagos National Park Directorate and Ecuadorian laws.

On September 3, 2019, the Galapagos National Park officials, in collaboration with the Ecuadorian Navy, intercepted five illegal fishing boats within the limits of the GMR. These vessels were detained and transported to the nearest port, Puerto Baquerizo Moreno, on the San Cristobal Island. After an identification of the captured individuals, it was determined that there were over 300 individuals of *Alopias* spp. among the five illegal boats, some of which could not be identified down to species level or maturity size due to their condition. For this study, 33 individuals of *A. pelagicus* and 21 of *A. superciliosus* were selected based on their preservation state, specifically selecting those that had a complete head and tail fin. Sex was recorded and total length (TL) data were taken to estimate precaudal length (PCL) based on established relationships. Finally, the first dorsal vertebra of each individual was collected. All remaining shark materials were then destroyed, as required by the Ecuadorian laws.

### Sample processing

The cleaning process for vertebrae involved the use of solvents to remove the neural arch and connective tissue. Subsequently, the vertebrae were sanded and polished before being placed in paper bags for drying. Each bag was labeled and stored in the Galapagos Science Center of the Universidad San Francisco de Quito. The diameter of each vertebrae was measured to determine its radius, which allowed the establishment of maturity stages (*i.e*., neonate, juvenile and adult) based on age and growth information developed by Liu et al. (1999) for *A. pelagicus* and Liu, Chiang & Chen (1998) for *A. superciliosus*. Subsequently, samples of vertebral collagen were collected from each growth section, using a micro drill and a 0.7 mm drill bit. Three sets of collagen samples were collected from each vertebra, provided that the individual had reach sexual maturity. However, if the individual was a juvenile, only two samples were obtained. In the case of *A. pelagicus*, 23 samples were gathered from mature adults and 14 from juveniles, whereas for *A. superciliosus*, 13 samples were obtained from adults and eight from juveniles.

Vertebral collagen samples were transferred to the Stable Isotope Biochemistry Laboratory of the Andalusian Institute of Earth Sciences IACT (CSIC-URG) in Granada, Spain, where they were weighed using an analytical balance to ensure precision. Samples weighing between 0.6 and 1 mg were stored in silver capsules and subjected to a hydrochloric acid steam bath to degrade any inorganic carbonates that could contaminate the analysis. This process was performed in a desiccator for 24 h. After being treated with the acid, the silver capsules were taken to the isotope ratio mass spectrometer (DELTA plus XL; Thermo Finnagen, Waltham, MA, USA) with 0.1‰ error, in order to quantify the δ^13^C and δ^15^N values. The results, expressed in parts per thousand (‰), were obtained using the equation: δ^13^C or δ^15^N = 1000([R_sample_/R_standard_] − 1), in which R_sample_ and R_standard_ are the ^13^C/^12^C or ^15^N/^14^N ratios of the sample and the standard, respectively. The standards used were Pee Dee Belemnite for δ^13^C and atmospheric N_2_ for δ^15^N. Finally, we measured the weight percentage of carbon and nitrogen concentrations in each sample and used the C/N ratio as a proxy for protein content (Logan et al., 2008).

### Data analysis

Shapiro-Wilk, Kolmogorov-Smirnov, Bartlett and Levene’s tests were employed to assess the normality and homogeneity of the data for both species. Then, Welch’s t-test, Student’s t-test, ANOVA, and Tukey’s test were applied to determine differences between species, sexes, and maturity stages. All statistical analyses were performed in R software using a significance level of *p* = 0.05.

The Bayesian standard ellipse areas (SEA) were used to estimate isotopic niche width and overlap among thresher sharks’ groups using the package SIBER (Stable Isotope Bayesian Ellipses in statistical software R) (Jackson et al., 2011). This Bayesian method provides a measure of the isotopic niche area at the population level, expressed as the SEA in units of area (‰^2^) and contains 95% of the data for each analysed group. Monte Carlo simulations were employed to correct the bivariate ellipses (δ^13^C and δ^15^N) surrounding the data points in the 95% confidence interval for the distributions of both isotopes (Jackson et al., 2011). The magnitude of the isotopic overlap (‰^2^) among species, sex and life stages were estimated using the estimations of the ellipses *via* maximum probability methods (Jackson et al., 2011).

## Results

The maximum length recorded for *A. pelagicus* and *A. superciliosus* were 2.99 and 2.86m respectively, while the C/N ratio ranged from 3.22‰ to 3.56‰ (Table 1), corroborating that the signatures were within the theoretical range established for the assimilation of protein (McConnaughey & McRoy, 1979).

**Table 1:** δ^13^C and δ^15^N signatures (expressed as ‰; mean ± SD, and C/N relation) of *A. pelagicus* and *A. superciliosus* in the Galapagos Marine Reserve.

Species	Sex/Life stage	δ^13^C (mean ± SD)	δ^15^N (mean ± SD)	C/N
*A. pelagicus*		−14.7 ± 1.18‰	9.5 ± 1.18‰	3.44
	Females	−14.5 ± 0.8‰	9.9 ± 0.7‰	3.49
	Males	−14.7 ± 0.7‰	9.5 ± 1.2‰	3.34
	Neonates	−15.0 ± 1.0‰	9.3 ± 1.0‰	3.56
	Juveniles	−14.8 ± 0.5‰	9.5 ± 1.3‰	3.43
	Adults	−14.3 ± 0.5‰	9.7 ± 1.1‰	3.22
*A. superciliosus*		−14.9 ± 1.6‰	8.0 ± 1.31‰	3.56
	Females	−14.3 ± 0.4‰	7.6 ± 1.4‰	3.40
	Males	−14.6 ± 1.0‰	8.1 ± 1.3‰	3.47
	Neonates	−14.5 ± 1.2‰	8.6 ± 0.8‰	3.53
	Juveniles	−14.6 ± 0.7‰	7.8 ± 1.4‰	3.49
	Adults	−14.4 ± 0.7‰	7.7 ± 1.4‰	3.41

**Note:**

Isotopic signatures are categorized by sex and life stage in both species.

### Isotopic comparison between species, sex and maturity stages

All isotopic values of both species were analyzed (Table 1), and interspecific differences were observed in δ^15^N signatures (t = 6.4, df = 98.07, *p* < 0.001) but not in δ^13^C signatures (t = −1.2, df = 94.58, *p* = 0.23). No differences were found in the δ^15^N and δ^13^C signatures between sexes for both species (*p* > 0.05). However, intraspecific differences were observed in δ^13^C signatures between maturity stages of *A. pelagicus* (F (2.73) = 4.61, *p* = 0.01), but not in δ^15^N signatures (F (2.73) = 0.44, *p* = 0.64). For the maturity stages of *A. superciliosus* there were no differences in δ^13^C (F (2.48) = 0.32, *p* = 0.72) and δ^15^N (F (2.48) = 2.42, *p* = 0.10) signatures. Given the dissimilarities in the δ^13^C signatures of the maturity stages of *A. pelagicus*, Tukey’s test was performed; showing that there are differences between the neonate *vs*. adult stages (−0.65‰, *p* = 0.01), and between juvenile *vs*. adult stages (−0.5‰, *p* = 0.04).

### Isotopic niche width and overlap

**Figure 2:**
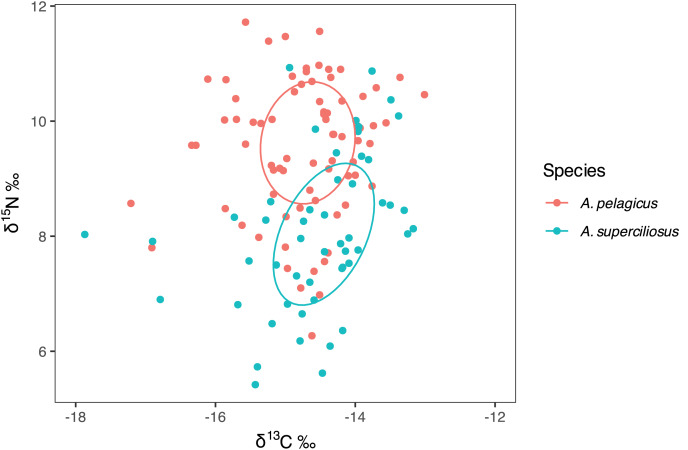
Isotopic niche area (δ^13^C and δ^15^N) of *A. pelagicus* and *A. superciliosus* in the Galapagos Marine Reserve.

**Figure 3:**
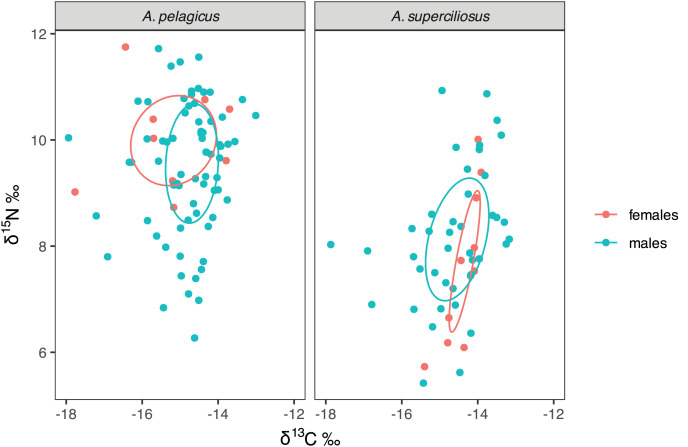
Isotopic niche area (δ^13^C and δ^15^N) of *A. pelagicus* and *A. superciliosus* in the Galapagos Marine Reserve. The ellipses area shows the degree of overlap within sex groups.

The SIBER test showed an isotopic niche overlap of 46% between both species (Fig. 2), which can be considered moderate, since *A. pelagicus* showed higher δ^15^N values than *A. superciliosus* (Fig. 2). A similar pattern was identified between sexes in both species (Fig. 3), where there was a 44% overlap between females and males of *A. pelagicus* was observed, while the overlap between females and males of *A. superciliosus* was 31%. However, the corrected standard ellipse (SEAc) of females of *A. pelagicus* was wider (5.3‰^2^) than that of males (3.3‰^2^) (Fig. 3 and Table 2), demonstrating some trophic flexibility in females. For *A. superciliosus* it was the opposite, with females showing a narrower isotopic niche (1.4‰^2^) compared to males (3.9‰^2^) (Fig. 3 and Table 2).

**Table 2:** Total isotopic area (TA‰^2^), standard ellipse area (SEA‰^2^) and corrected standard ellipse (SEAc‰^2^) recorded for *A. pelagicus* and *A. superciliosus* in the Galapagos Marine Reserve.

Species	Sex/Life stage	TA (‰^2^)	SEA (‰^2^)	SEAc (‰^2^)
*A. pelagicus*				
	Females	9.8	4.7	5.3
	Males	15.9	3.3	3.4
	Neonates	32.2	10.1	10.4
	Juveniles	15.7	3.8	4.0
	Adults	6.0	1.9	2.0
*A. superciliosus*				
	Females	2.3	1.3	1.4
	Males	15.8	3.8	3.9
	Neonates	14.8	5.5	5.8
	Juveniles	10.0	3.2	3.4
	Adults	7.1	3.2	3.3

**Note:**

Values are categorized by sex and life stage in both species.

**Figure 4:**
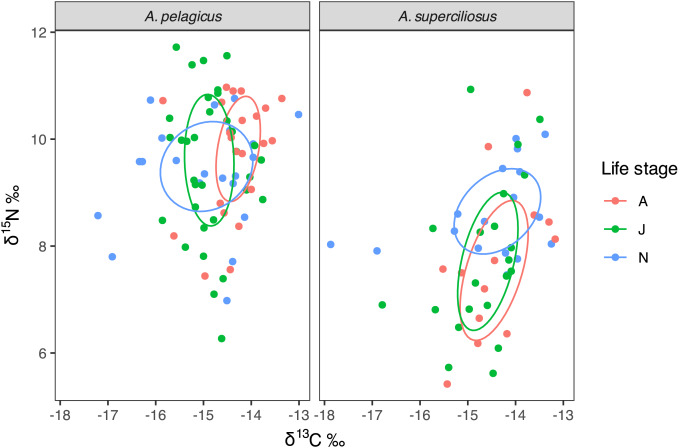
Isotopic niche area (δ^13^C and δ^15^N) of *A. pelagicus* and *A. superciliosus* in the Galapagos Marine Reserve. The ellipses area shows the degree of overlap within age categories in both species.

Bayesian ellipses demonstrate that neonates of *A. pelagicus* exhibited a 54% overlap with respect to juveniles and a 33% overlap with adults, whereas juveniles and adults shared an overlap of 45% (Fig. 4). In contrast, the neonates of *A. superciliosus* displayed a 47% overlap with juveniles and a 40% overlap with adults, while juveniles and adults had an 80% overlap (Fig. 4). Finally, corrected standard ellipse (SEAc) of all age categories in both species, showed that the isotopic niche of neonates is broader than that of juvenile and adults (Table 2).

## Discussion

### Feeding patterns of thresher shark species

The δ^13^C and δ^15^N signatures suggests a consistent pattern of habitat use by *A. pelagicus* in the GMR throughout their life. This species is known to prefer offshore ecosystems, making its δ^13^C signatures depleted (Polo-Silva et al., 2013). The populations of *A. pelagicus* inhabiting mainland Ecuador have more impoverished δ^13^C signatures (~2‰) than those inhabiting the GMR (Polo-Silva et al., 2013; Páez-Rosas et al., 2018), suggesting the use from oceanic areas where there is less supply of nutrients. Although the populations of *A. pelagicus* that inhabit the GMR could have a more coastal strategy, it must also be considered that the shorelines of archipelago are characterized by having mangrove ecosystems (Moity, Delgado & Salinas-de-León, 2019). In general, mangrove-dominated shorelines are rich in suspended particulate matter, dissolved organic matter and nutrients (Cawley et al., 2012), which could be enriching the δ^13^C signatures of *A. pelagicus* in the GMR, and in turn favoring its permanence in this region.

The δ^15^N signatures of *A. pelagicus* at the mainland Ecuador and GMR do not present major differences (Polo-Silva et al., 2013), so both populations could be consuming similar prey throughout the equatorial Pacific. The diet of *A. pelagicus* in mainland Ecuador consists of three main prey: red flying squid, *Ommastrephes bartramii*; Humboldt squid, *Dosidicus gigas*; and purpleback flying squid, *Sthenoteuthis oualaniensis* (Calle-Morán & Galván-Magaña, 2020), species that are widely distributed throughout the Eastern Tropical Pacific (Galván-Magaña et al., 2013; Yu, Chen & Liu, 2021). Typically, *A. pelagicus* migrates vertically during the day, spending most of the daylight period at 200–300 m in the mesopelagic zone, and most of the night at 50–150 m in the epipelagic zone (Andrzejaczek et al., 2022). This vertical migration pattern suggests that *A. pelagicus* may actively pursue prey (*i.e*., during the squid’s dial migrations) (González-Pestana et al., 2019), however, It is possible that also feeds during the day at greater depths, following prey to their deeper locations (Arostegui et al., 2020).

There were no isotopic differences between males and females of *A. pelagicus*, suggesting that both sexes preferred similar habitats and feeding sources. This finding coincides with previous studies conducted on this species in Ecuadorian Pacific (Polo-Silva et al., 2013; Páez-Rosas et al., 2018), as well on the west coast of the Baja California Peninsula, Mexico (Sánchez-Latorre et al., 2023). However, our results showed a moderate overlap between sexes, where females had broader isotopic niches, so they could be using other habitats unlike males. This condition could be related to the fact that females need to expand their feeding areas to consume a high amount of key nutrients, such as fatty acids and proteins (Liu et al., 1999), that are crucial for reaching sexual maturity and maintaining the energy required for gestation (Liu et al., 1999). This species also exhibits ovofagia, a process that directs its energy to produce fewer but larger and more mature embryos, which decreases the risk of predation (Miller, Wails & Sulikowski, 2022). Therefore, this reproductive strategy requires a lot of energy, which explains why *A. pelagicus* females consume prey from different habitats, such as deep-sea squids (Polo-Silva et al., 2013).

Prior research has examined the diet of *A. superciliosus* in the Ecuadorian Pacific (Polo-Silva et al., 2007; Polo-Silva, Rendón & Galván-Magaña, 2009). However, these studies were conducted in mainland waters, making the current research in baseline information for the GMR. Our isotopic signatures suggest the exploitation of inshore habitats with high productivity within the GMR. A characteristic that coincides with the dietary references of *A. superciliosus*, where they mention the consumption of benthic and coastal fish (Polo-Silva et al., 2007; Polo-Silva, Rendón & Galván-Magaña, 2009). Thus, based on isotopic signatures of both *Alopias* species we can infer that there is no interspecific competition, despite using the same habitat within the GMR. This condition would be supported by the Bayesian ellipses, which demonstrated a moderate overlap between both species. Therefore, the consumption of squids by *A. pelagicus* would cause it to migrate frequently to pelagic ecosystems to feed (Polo-Silva et al., 2013; Calle-Morán & Galván-Magaña, 2020), while *A. superciliosus* would look for more coastal food sources (Polo-Silva et al., 2007; Polo-Silva, Rendón & Galván-Magaña, 2009).

There were also no isotopic differences were found between males and females of *A. superciliosus*, indicating a similar use of food sources. Polo-Silva et al. (2007) established that female *A. superciliosus* remain in offshore areas, but can migrate to inshore ecosystems to feed, while males remain in coastal areas due to a more specialized diet. Stomach content analysis has revealed a more diverse diet in females (22 prey) compared to males (13 prey); where both sexes have preference for demersal fish, however, female preferences also extended to deep-water squids (Polo-Silva et al., 2007; Polo-Silva, Rendón & Galván-Magaña, 2009). Therefore, it is likely that *A. superciliosus* females migrate to offshore ecosystems more frequently than males, particularly during gestation and parturition periods. The isotopic niche had a low overlap between sex, confirming a differential pattern in habitat use and potential prey. This could imply that although females and males coexist in the same environment, they maintain varied feeding patterns as a strategy to prevent potential intraspecific competition (Briones-Mendoza, Carrasco-Puig & Toala-Franco, 2021).

### Ontogenetic feeding patterns

Neonates of *A. pelagicus* exhibited a more extensive isotopic niche breadth than other groups. The observed variation in δ^13^C may reflect not only the environmental conditions experienced by the neonates but also the maternal signal or a combination of influences. It is generally challenging to assess δ^13^C and δ^15^N signatures in the early life stages of sharks, unless the rate of maternal isotopic signal loss is measured for each species (Olin et al., 2011; Broadhurst et al., 2019). However, there is evidence that at 1 year of age they would have already lost their mother’s δ^13^C and δ^15^N signatures, reflecting a condition of independent consumer (Olin, Shipley & McMeans, 2018; Páez-Rosas et al., 2021).

Juvenile sharks exhibited a more restricted habitat usage than neonates, which indicates a shift from opportunists’ habits in neonates to more specialized habits as they grow. Juvenile sharks prioritize meeting their high energy demands and invest this energy in growing at a high rate to reach sexual maturity (Bethea, Buckel & Carlson, 2004; Crear et al., 2021). However, in early stages, pelagic sharks do not yet exhibit specific discrimination between prey, so they could consume coastal prey from lower trophic levels (*e.g*., mollusks and arthropods) and higher ones (*e.g*., pelagic and benthic fishes) (Bethea, Buckel & Carlson, 2004). Studies on the identification of juvenile sharks in the GMR have revealed that *A. pelagicus* is typically found near coasts and bays, always as solitary individuals in apparent feeding activities (Llerena-Martillo, Peñaherrera-Palma & Espinoza, 2018).

Adult and juvenile *A. pelagicus* demonstrated remarkably similar isotopic averages, indicating comparable habitat use and prey consumption patterns. However, when evaluating the isotopic niche areas, juveniles exhibited a larger amplitude compared to adults. This difference is attributed to the consumption of lower trophic level prey by juveniles and their more generalized habits compared to adults (Polo-Silva et al., 2013; Sánchez-Latorre et al., 2023). In adults the reproduction becomes the most important energetic target (Sims, 2005), so physiological adaptations of *A. pelagicus* also change as they grow, allowing them to exploit deeper ecosystems (Andrzejaczek et al., 2022). This condition allows them to prey at higher trophic levels, such as cephalopods (*O. bartramii, D. gigas*, and *S. oualaniensis*), which comprise 90% of the diet of adult *A. pelagicus* from the Equatorial Pacific (Galván-Magaña et al., 2013; Calle-Morán & Galván-Magaña, 2020).

No isotopic differences were detected among maturity stages (*i.e*., neonates, juveniles, and adults) in *A. superciliosus*, however, isotopic niche areas suggest different feeding patterns for each stage. Neonates of *A. superciliosus* exhibited a wide isotopic niche indicating the utilization of offshore and inshore habitats. However, this isotopic niche breadth may be related to the isotopic contribution of the mother, which suggests that neonates’ isotopic signatures of *A. superciliosus* are affected by maternal inputs, even after the first year of life (Hussey, MacNeil & Fisk, 2010; Olin et al., 2011).

Juveniles displayed a reduction in isotopic niche breadth associated with a change in their feeding patterns, specifically in inshore environments. In contrast to the neonate stage where the primary goal is to avoid predators, the juvenile stage is characterized by a high growth rate (Branstetter, 1990). Therefore, this greater need for energy leads them to consume high-calorie prey such as the coastal fish *L. argentus* and *M. gayi* (Polo-Silva et al., 2007; Polo-Silva, Rendón & Galván-Magaña, 2009). Juveniles of *A. superciliosus* spend more time foraging in shallow water during the day and in deeper water at night, whereas adults engage in the reverse pattern (Fernandez-Coelho et al., 2015). Therefore, it is possible that the difference in the use of shallow and deep habitats (Fernandez-Coelho et al., 2015) is a strategy for avoiding potential competition between individuals of the same species.

The juvenile and adult stages of *A. superciliosus* revealed a high degree of overlap, this finding suggests that individuals of different sizes and sexes share the similar habitats. However, as *A. superciliosus* progresses through different maturity stages, its feeding strategies become increasingly specialized and focused on inshore habitats (Preti et al., 2008). Even research on vertical migration of this species has shown that adults can dive to depths up to 700 m, where they complement their diet with myctophids and squids (Nakano et al., 2003; Coelho, Fernandez-Carvalho & Santos, 2015).

## Conclusions

Lamniformes are known to be present in coastal and oceanic ecosystems. However, unlike *A. pelagicus* and *A. vulpinus*, *A. superciliosus* inhabits continental slopes, which distinguishes it from other *Alopias* species (Estrada et al., 2003; Smith et al., 2008). The outcomes of this research contribute to the knowledge on the trophic ecology of thresher sharks in the GMR and provide data to establish their contribution within the ecological dynamics of this region. However, it is challenging to accurately assess the status of *Alopias* populations in the Ecuadorian Pacific, and to develop effective management strategies for this species. Therefore, it is crucial to continue generating information to develop sustainable fisheries and effective conservation measures for these populations.

## Supplemental Information

10.7717/peerj.18681/supp-1 Supplemental Information 1 Dataset.

## Additional Information and Declarations

### Data Availability

The following information was supplied regarding data availability: The raw data is available in the Supplemental Files.

## References

[ref-1] Acuña-Marrero D, Smith AN, Salinas-de-León P, Harvey ES, Pawley MD, Anderson MJ (2018). Spatial patterns of distribution and relative abundance of coastal shark species in the Galapagos Marine Reserve. Marine Ecology Progress Series.

[ref-2] Adams TS, Sterner RW (2000). The effect of dietary nitrogen content on trophic level 15N enrichment. Limnology and Oceanography.

[ref-3] Andrzejaczek S, Lucas TC, Goodman MC, Hussey NE, Armstrong A, Carlisle A, Coffey DM, Gleiss AC, Huveenirs C, Jocoby DM, Meekan MG, Mourier J, Peel LR, Abrantes K, Afonso AS, Ajemian MJ, Anderson BN, Anderson SD, Araujo G, Armstrong AO, Bach P, Barnett AB, Bennett MB, Bezerra NA, Bonfil R, Boustany AM, Bowlby HD, Branco I, Braun CD, Brooks EJ, Brown J, Burke PJ, Butcher P, Castleton M, Chapple TK, Chateau O, Clarke M, Coelho R, Cortes E, Couturier LE, Cowley PD, Croll DA, Cuevas JM, Curtis TH, Dagorn L, Dale JJ, Daly R, Dewar H, Doherty PD, Domingo A, Dove AD, Drew M, Dudgeon CL, Duffy CA, Elliott RG, Ellis JR, Erdmann MV, Farrugia TJ, Ferreira LC, Ferretti F, Filmalter JD, Finucci B, Fischer C, Fitzpatrick R, Forget F, Forsberg K, Francis MP, Franks BR, Gallagher AJ, Galvan-Magana F, García ML, Gaston TF, Gillanders BM, Gollock MJ, Green JR, Green S, Griffiths CA, Hammerschlag N, Hasan A, Hawkes LA, Hazin F, Heard M, Hearn A, Hedges KJ, Henderson SM, Holdsworth J, Holland KN, Howey LA, Hueter RE, Humphries NE, Hutchinson M, Jaine FA, Jorgensen SA, Kanive PE, Labaja J, Lana FO, Lassauce H, Lipscombe RS, Llewellyn F, Macena BC, Mambrasar R, McAllister JD, Phillips SR, McGregor F, McMillan MN, McNaughton LM, Mendonça SA, Meyer CG, Meyers M, Mohan JA, Montgomery JC, Mucientes G, Musyl MK, Nasby-Lucas M, Natanson LJ, O’Sullivan JB, Oliveira P, Papastamtiou YP, Patterson TA, Pierce SJ, Queiroz N, Radford CA, Richardson AJ, Richardson AJ, Righton D, Rohner CA, Royer MA, Saunders RA, Schaber M, Schallert RJ, Scholl MC, Seitz AC, Semmens JM, Setyawan E, Shea BD, Shidqi RA, Shillinger GL, Shipley ON, Shivji M, Sianipar AB, Silva JF, Sims DW, Skomal GV, Sousa LL, Southall EJ, Spaet JL, Stehfest KM, Stevens G, Stewart JD, Sulikowski JA, Syakurachman I, Thorrold SR, Thums M, Tickler D, Tolloti MT, Townsend KA, Travassos P, Tyminski JP, Vaudo JJ, Veras D, Wantiez L, Weber SB, Wells RJD, Weng KC, Wetherbee BM, Williamson JE, Witt MJ, Wright S, Zilliacus K, Block BA, Curnick DJ (2022). Diving into the vertical dimension of elasmobranch movement ecology. Science Advances.

[ref-4] Arostegui MC, Gaube P, Berumen ML, DiGiulian A, Jones BH, Røstad A, Braun CD (2020). Vertical movements of a pelagic thresher shark (*Alopias pelagicus*): insights into the species’ physiological limitations and trophic ecology in the Red Sea. Endangered Species Research.

[ref-5] Baum JK, Myers RA (2004). Shifting baselines and the decline of pelagic sharks in the Gulf of Mexico. Ecology Letters.

[ref-6] Baum JK, Worm B (2009). Cascading top-down effects of changing oceanic predator abundances. Journal of Animal Ecology.

[ref-7] Bearhop S, Adams CE, Waldron S, Fuller RA, Macleod H (2004). Determining trophic niche width: a novel approach using stable isotope analysis. Journal of Animal Ecology.

[ref-8] Bethea DM, Buckel JA, Carlson JK (2004). Foraging ecology of the early life stages of four sympatric shark species. Marine Ecology Progress Series.

[ref-9] Bird CS, Veríssimo A, Magozzi S, Abrantes KG, Aguilar A, Al-Reasi H, Barnett A, Bethea DM, Biais G, Borrell A, Bouchoucha M, Boyle M, Brooks EJ, Brunnschweiler J, Bustamante P, Carlisle A, Catarino D, Caut S, Cherel Y, Chouvelon T, Churchill D, Ciancio J, Claes J, Colaço A, Courtney DL, Cresson P, Daly R, Necker L, Endo T, Figueiredo I, Frisch AJ, Hansen JH, Heithaus M, Hussey NH, Iitembu J, Juanes F, Kinney MJ, Kiszka JJ, Klarian SA, Kopp D, Leaf R, Li Y, Lorrain A, Madigan DJ, Maljković A, Malpica-Cruz L, Matich P, Meekan MG, Ménard F, Menezes GM, Munroe SE, Newman MC, Papastamatiou YP, Pethybridge H, Plumlee JD, Polo-Silva C, Quaeck-Davies K, Raoult V, Reum J, Torres-Rojas YH, Shiffman DS, Shipley ON, Speed CW, Staudinger MD, Teffer AK, Tilley A, Valls M, Vaudo JJ, Wai T, Wells RD, Wyatt AS, Yool A, Trueman CN (2018). A global perspective on the trophic geography of sharks. Nature Ecology & Evolution.

[ref-10] Branstetter S (1990). Early life-history implications of selected carcharhinoid and lamnoid sharks of the northwest Atlantic. https://scholarworks.wm.edu/vimsbooks/40.

[ref-11] Briones-Mendoza J, Carrasco-Puig P, Toala-Franco D (2021). Reproductive biology aspects of *Alopias pelagicus* and *A. superciliosus* (Lamniformes: Alopiidae) in the Ecuadorian Pacific. Neotropical Ichthyology.

[ref-12] Briones-Mendoza J, Mejía D, Carrasco-Puig P (2022). Catch composition, seasonality, and biological aspects of sharks caught in the Ecuadorian Pacific. Diversity.

[ref-13] Broadhurst MK, Domit C, Trevizani TH, Raoult V, Millar RB (2019). Mother-embryo isotope fractionation in the pygmy devilray *Mobula kuhlii* cf. *eregoodootenkee*. Journal of Fish Biology.

[ref-14] Calle-Morán MD, Galván-Magaña F (2020). Diet composition and feeding habits of the pelagic thresher shark *Alopias pelagicus* in Eastern Central Pacific Ocean, Ecuadorian waters. Journal of the Marine Biological Association of the United Kingdom.

[ref-15] Campana SE, Natanson LJ, Myklevoll S (2002). Bomb dating and age determination of large pelagic sharks. Canadian Journal of Fisheries and Aquatic Sciences.

[ref-16] Carlisle AB, Goldman KJ, Litvin SY, Madigan DJ, Bigman JS, Swithenbank AM, Block BA (2015). Stable isotope analysis of vertebrae reveals ontogenetic changes in habitat in an endothermic pelagic shark. Proceedings of the Royal Society B: Biological Sciences.

[ref-17] Cawley KM, Ding Y, Fourqurean J, Jaffé R (2012). Characterizing the sources and fate of dissolved organic matter in Shark Bay, Australia: a preliminary study using optical properties and stable carbon isotopes. Marine and Freshwater Research.

[ref-18] Cerutti-Pereyra F, Salinas-De-León P, Arnés-Urgellés C, Suarez-Moncada J, Espinoza E, Vaca L, Páez-Rosas D (2022). Using stable isotopes analysis to understand ontogenetic trophic variations of the scalloped hammerhead shark at the Galapagos Marine Reserve. PLOS ONE.

[ref-19] Coelho R, Fernandez-Carvalho J, Santos MN (2015). Habitat use and diel vertical migration of bigeye thresher shark: overlap with pelagic longline fishing gear. Marine Environmental Research.

[ref-20] Compagno LJV (1984). FAO species catalog. Vol 4. Sharks of the world: an annotated and illustrated catalog of shark species known to date. Part 1. Hexanchiformes to Lamniformes. FAO Fisheries Synopsis.

[ref-21] Crear DP, Peterson CD, Higgs JM, Hendon JM, Hoffmayer ER (2021). Ontogenetic habitat partitioning among four shark species within a nursery ground. Marine and Freshwater Research.

[ref-22] Edgar GJ, Banks S, Bensted-Smith R, Calvopiña M, Chiriboga A, Garske LE, Salazar S (2008). Conservation of threatened species in the Galapagos Marine Reserve through identification and protection of marine key biodiversity areas. Aquatic Conservation: Marine and Freshwater Ecosystems.

[ref-23] Estrada JA, Rice AN, Lutcavage ME, Skomal GB (2003). Predicting trophic position in sharks of the north-west Atlantic Ocean using stable isotope analysis. Journal of the Marine Biological Association of the United Kingdom.

[ref-24] Estrada JA, Rice AN, Natanson LJ, Skomal GB (2006). Use of isotopic analysis of vertebrae in reconstructing ontogenetic feeding ecology in white sharks. Ecology.

[ref-25] Estupiñán-Montaño C, Galván-Magaña F, Sánchez-González A, Elorriaga-Verplancken FR, Delgado-Huertas A, Páez-Rosas D (2019). Dietary ontogeny of the blue shark *Prionace glauca*, based on the analysis of δ^13^C and δ^15^N in vertebrae. Marine Biology.

[ref-26] Fernandez-Coelho J, Coelho R, Mejuto J, Cortés E, Domingo A, Yokawa K, Liu K, García-Cortés B, Forselledo R, Ohshimo S, Ramos-Cartelle A, Tsai W, Santos MN (2015). Pan-Atlantic distribution patterns and reproductive biology of the bigeye thresher, *Alopias superciliosus*. Reviews in Fish Biology and Fisheries.

[ref-27] Ferretti F, Worm B, Britten GL, Heithaus MR, Lotze HK (2010). Patterns and ecosystem consequences of shark declines in the ocean. Ecology Letters.

[ref-28] Forryan A, Naveira Garabato AC, Vic C, Nurser AG, Hearn AR (2021). Galápagos upwelling driven by localized wind-front interactions. Scientific Reports.

[ref-29] France RL (1995). Carbon-13 enrichment in benthic compared to planktonic algae: Foodweb implications. Marine Ecology Progress Series.

[ref-30] Galván-Magaña F, Polo-Silva C, Hernández-Aguilar SB, Sandoval-Londoño A, Ochoa-Díaz MR, Aguilar-Castro N, Abitia-Cárdenas LA (2013). Shark predation on cephalopods in the Mexican and Ecuadorian Pacific Ocean. Deep Sea Research Part II: Topical Studies in Oceanography.

[ref-31] González-Pestana A, Acuña-Perales N, Córdova F, Coasaca J, Alfaro E, Alfaro-Shigueto J, Mangel JC (2019). Feeding habits of thresher sharks *Alopias sp*. in northern Peru: predators of Humboldt squid (*Dosidicus gigas*). Journal of the Marine Biological Association of the United Kingdom.

[ref-32] Heupel MR, Carlson JK, Simpfendorfer CA (2007). Shark nursery areas: concepts, definition, characterization and assumptions. Marine Ecology Progress Series.

[ref-33] Heylings P, Bensted-Smith R, Altamirano M (2002). Zonificación e historia de la Reserva Marina de Galápagos. Reserva Marina de Galápagos.

[ref-34] Hussey NE, MacNeil MA, Fisk AT (2010). The requirement for accurate diet-tissue discrimination factors for interpreting stable isotopes in sharks: comment on: stable isotope dynamics in elasmobranch fishes. Hydrobiologia.

[ref-35] Hussey NE, MacNeil MA, Olin JA, McMeans BC, Kinney MJ, Chapman DD, Fisk AT (2012). Stable isotopes and elasmobranchs: tissue types, methods, applications and assumptions. Journal of Fish Biology.

[ref-36] Jackson A, Inger R, Parnell A, Bearhop S (2011). Comparing isotopic niche widths among and within communities: SIBER—stable isotope Bayesian ellipses. Journal of Animal Ecology.

[ref-37] Liu KM, Chen CT, Liao TH, Joung SJ (1999). Age, growth, and reproduction of the pelagic thresher shark, *Alopias pelagicus* in the Northwestern Pacific. Copeia.

[ref-38] Liu KM, Chiang PJ, Chen CT (1998). Age and growth estimates of the bigeye thresher shark, *Alopias superciliosus*, in northeastern Taiwan waters. Fishery Bulletin.

[ref-39] Llerena-Martillo Y, Peñaherrera-Palma C, Espinoza E (2018). Fish assemblages in three fringed mangrove bays of Santa Cruz Island, Galapagos marine reserve. Revista Biología Tropical.

[ref-40] Logan J, Jardine T, Miller TJ, Bunn S, Cunjak R, Lutcavage M (2008). Lipid corrections in carbon and nitrogen stable isotope analyses: comparison of chemical extraction and modelling methods. Journal of Animal Ecology.

[ref-41] MacNeil MA, Skomal GB, Fisk AT (2005). Stable isotopes from multiple tissues reveal diet switching in sharks. Marine Ecology Progress Series.

[ref-42] Martínez-Ortíz J, Aires-da-Silva AM, Lennert-Cody CE, Maunder MN (2015). The Ecuadorian artisanal fishery for large pelagics: species composition and spatio-temporal dynamics. PLOS ONE.

[ref-43] McConnaughey T, McRoy C (1979). Food-web structure and fractionation of carbon isotopes in the Bering Sea. Marine Biology.

[ref-44] Michener RH, Schell DM, Lajtha K, Michener RH (1994). Stable isotope ratios as tracers in marine and aquatic food webs. Stable Isotopes in Ecology and Environmental Science.

[ref-45] Miller E, Wails CN, Sulikowski J (2022). It’s a shark-eat-shark world, but does that make for bigger pups? A comparison between oophagous and non-oophagous viviparous sharks. Reviews in Fish Biology and Fisheries.

[ref-46] Moity N, Delgado B, Salinas-de-León P (2019). Mangroves in the Galapagos Islands: distribution and dynamics. PLOS ONE.

[ref-47] Nakano H, Matsunaga H, Okamoto H, Okazaki M (2003). Acoustic tracking of bigeye thresher shark Alopias superciliosus in the eastern Pacific Ocean. Marine Ecology Progress Series.

[ref-48] Newsome SD, Martinez del Rio C, Bearhop S, Phillips DL (2007). A niche for isotopic ecology. Frontiers in Ecology and the Environment.

[ref-49] Olin JA, Hussey NE, Fritts M, Heupel MR, Simpfendorfer CA, Poulakis GR, Fisk AT (2011). Maternal meddling in neonatal sharks: implications for interpreting stable isotopes in young animals. Rapid Communications in Mass Spectrometry.

[ref-50] Olin JA, Shipley ON, McMeans BC (2018). Stable isotope fractionation between maternal and embryo tissues in the Bonnethead shark (*Sphyrna tiburo*). Environmental Biology of Fishes.

[ref-51] Oliver SP, Turner JR, Gann K, Silvosa M, D’Urban Jackson T, Tsikliras AC (2013). Thresher sharks use tail-slaps as a hunting strategy. PLOS ONE.

[ref-52] Páez-Rosas D, Insuasti-Zarate P, Riofrío-Lazo M, Galván-Magaña F (2018). Feeding behavior and trophic interaction of three shark species in the Galapagos Marine Reserve. PeerJ.

[ref-53] Páez-Rosas D, Suarez-Moncada J, Arnes-Urgelles C, Espinoza E, Robles Y, Salinas-De-León P (2024). Assessment of nursery areas for the scalloped hammerhead shark (*Sphyrna lewini*) across the Eastern Tropical Pacific using a stable isotopes approach. Frontiers in Marine Science.

[ref-54] Páez-Rosas D, Suarez-Moncada J, Elorriaga-Verplancken FR, Proaño A, Arnés-Urgellés C, Salinas-de-León P, Galván-Magaña F (2021). Trophic variation during the early stages of blacktip sharks (*Carcharhinus limbatus*) within coastal nurseries of the Galapagos Marine Reserve. Journal of Sea Research.

[ref-55] Palacios DM, Bograd SJ, Foley DG, Schwing FB (2006). Oceanographic characteristics of biological hot spots in the North Pacific: a remote sensing perspective. Deep Sea Research Part II: Topical Studies in Oceanography.

[ref-56] Polo-Silva C, Baigorrí-Santacruz Á, Galván-Magaña F, Grijalba-Bendeck M, Sanjuan-Muñoz A (2007). Hábitos alimentarios del tiburón zorro Alopias superciliosus (Lowe, 1839) en el Pacífico ecuatoriano. Revista de Biología Marina y Oceanografía.

[ref-57] Polo-Silva C, Newsome SD, Galván-Magaña F, Grijalba-Bendeck M, Sanjuan-Muñoz A (2013). Trophic shift in the diet of the pelagic thresher shark based on stomach contents and stable isotope analyses. Marine Biology Research.

[ref-58] Polo-Silva C, Rendón L, Galván-Magaña F (2009). Descripción de la dieta de los tiburones zorro (Alopias pelagicus) y (Alopias superciliosus) durante la época lluviosa en aguas ecuatorianas. Pan-American Journal of Aquatic Sciences.

[ref-59] Post DM (2002). Using stable isotopes to estimate trophic position: models, methods, and assumptions. Ecology.

[ref-60] Preti AP, Kohin S, Dewar H, Ramon D (2008). Feeding habits of the bigeye thresher (*Alopias superciliosus*) sampled from the California-based drift gillnet fishery. California Cooperative Oceanic Fisheries Investigations Reports.

[ref-61] Raharjo B, Hartati R, Redjeki S (2024). Population status of thresher shark listed in Appendix II CITES of Southern Java Seas. Indonesia Egyptian Journal of Aquatic Research.

[ref-62] Rigby CL, Barreto R, Carlson J, Fernando D, Fordham S, Francis MP, Herman K, Jabado RW, Liu KM, Marshall A, Pacoureau N, Romanov E, Sherley RB, Winker H (2019). Alopias pelagicus.

[ref-63] Salinas-de-León P, Acuña-Marrero D, Rastoin E, Friedlander AM, Donovan MK, Sala E (2016). Largest global shark biomass found in the northern Galápagos Islands of Darwin and Wolf. PeerJ.

[ref-64] Salinas-de-León P, Andrade S, Arnés-Urgellés C, Bermudez JR, Bucaram S, Buglass S, Worm B (2020). Evolution of the Galapagos in the Anthropocene. Nature Climate Change.

[ref-65] Salinas-de-León P, Fierro-Arcos D, Suarez-Moncada J, Proaño A, Guachisaca- Salinas J, Páez-Rosas D (2019). A matter of taste: Spatial and ontogenetic variations on the trophic ecology of the tiger shark at the Galapagos Marine Reserve. PLOS ONE.

[ref-66] Sánchez-Latorre C, Galván-Magaña F, Elorriaga-Verplancken FR, Tripp-Valdez A, González-Armas R, Piñón-Gimate A, Delgado-Huertas A (2023). Trophic ecology during the ontogenetic development of the pelagic thresher shark *Alopias pelagicus* in Baja California Sur, Mexico. Diversity.

[ref-67] Schaeffer BA, Morrison JM, Kamykowski D, Feldman GC, Xie L, Liu Y, Banks S (2008). Phytoplankton biomass distribution and identification of productive habitats within the Galapagos Marine Reserve by MODIS, a surface acquisition system, and in-situ measurements. Remote Sensing of Environment.

[ref-68] Sepulveda CA, Wegner NC, Bernal D, Graham JB (2005). The red muscle morphology of the thresher sharks (family Alopiidae). Journal of Experimental Biology.

[ref-69] Shen Y, Gong Y, Wu F, Li Y (2022). Retrospective stable isotopes of vertebrae reveal sexual ontogenetic patterns and trophic ecology in oceanic whitetip shark, *Carcharhinus longimanus*. Ecology and Evolution.

[ref-70] Shiffman DS, Macdonald CC, Wallace SS, Dulvy NK (2021). The role and value of science in shark conservation advocacy. Scientific Reports.

[ref-71] Sims DW, Ruckstuhl K, Neuhaus P (2005). Differences in habitat selection and reproductive strategies of male and female sharks. Sexual Segregation in Vertebrates.

[ref-72] Smith SE, Rasmussen RC, Ramon DA, Cailliet GM (2008). The biology and ecology of thresher sharks (Alopiidae). Sharks of the Open Ocean: Biology, Fisheries and Conservation.

[ref-73] Sweet WV, Morrison JM, Kamykowski D, Schaeffer BA, Banks S, McCulloch A (2007). Water mass seasonal variability in the Galápagos Archipelago. Deep Sea Research Part I: Oceanographic Research Papers.

[ref-74] Werner EE, Gilliam JF (1984). The ontogenetic niche and species interactions in size-structured populations. Annual Review of Ecology and Systematics.

[ref-75] Worm B, Orofino S, Burns ES, D’Costa NG, Manir Feitosa L, Palomares ML, Bradley D (2024). Global shark fishing mortality still rising despite widespread regulatory change. Science.

[ref-76] Yu W, Chen X, Liu L (2021). Synchronous variations in abundance and distribution of *Ommastrephes bartramii* and *Dosidicus gigas* in the Pacific Ocean. Journal of Ocean University of China.

